# Generalizability of Randomized Clinical Trial Outcomes for Diabetes Control Resulting From Bariatric Surgery

**DOI:** 10.1097/AS9.0000000000000414

**Published:** 2024-04-10

**Authors:** Edward H. Livingston, Hila Zelicha, Erik P. Dutson, Zhaoping Li, Matthew L. Maciejewski, Yijun Chen

**Affiliations:** * From the Department of Surgery, UCLA School of Medicine, Los Angeles, CA; † Division of Clinical Nutrition, UCLA School of Medicine, Los Angeles, CA; ‡ Department of Medicine, VA Greater Los Angeles Health System, Los Angeles, CA; § Center of Innovation to Accelerate Discovery and Practice Transformation (ADAPT), Durham Veterans Affairs Health Care System, Durham, NC; ‖ Department of Population Health Sciences, Duke University, Durham, NC; ¶ Division of General Internal Medicine, Department of Medicine, Duke University, Durham, NC.

**Keywords:** gastric sleeve resection, generalizability, heterogeneity, obesity, randomized clinical trial

## Abstract

**Objective:**

To assess the external validity of randomized controlled trials (RCTs) of bariatric surgical treatment on diabetes control.

**Background:**

Multisite RCTs provide the strongest evidence supporting clinical treatments and have the greatest internal validity. However, characteristics of trial participants may not be representative of patients receiving treatment in the real world. There is a need to assess how the results of RCTs generalize to all contemporary patient populations undergoing treatments.

**Methods:**

All patients undergoing sleeve gastrectomy at University of California Los Angeles (UCLA) between January 8, 2018 and May 19, 2023 had their baseline characteristics, weight change, and diabetes control compared with those enrolled in the surgical treatment and medications potentially eradicate diabetes efficiently (STAMPEDE) and diabetes surgery study (DSS) RCTs of bariatric surgery’s effect on diabetes control. Weight loss and diabetes control were compared between UCLA patients who did and did not fit the entry criteria for these RCTs.

**Results:**

Only 65 (17%) of 387 patients with diabetes fulfilled the eligibility criteria for STAMPEDE, and 29 (7.5%) fulfilled the criteria for DSS due to being older, having higher body mass index, and lower HbA1c. UCLA patients experienced slightly less weight loss than patients in the RCTs but had similar diabetes control. The 313 (81%) patients not eligible for study entry into either RCT had similar long-term diabetes control as those who were eligible for the RCTs.

**Conclusions:**

Even though only a very small proportion of patients undergoing bariatric surgery met the eligibility criteria for the 2 major RCTs, most patients in this contemporary cohort had similar outcomes. Diabetes outcomes from STAMPEDE and DSS generalize to most patients undergoing bariatric surgery for diabetes control.

## Abstract 2

Mini abstract:

Fewer than 17% of patients undergoing bariatric surgery for diabetes would have been eligible for enrollment in the major studies demonstrating its efficacy. This study shows that the results of the major randomized controlled trials generalize to all patients, even those who are very different.

## INTRODUCTION

Evidence for treatment effects with the greatest internal validity is derived from randomized controlled trials (RCTs). However, they often enroll patients not typical of the general population because of strict inclusion/exclusion criteria or are carried out in settings that differ from real-world practice, so the external validity (eg, generalizability) of RCTs is often questioned.^1^ Limited generalizability may lead to RCT results not being fully accepted as evidence of treatment benefit by policymakers, insurers, guideline committees, or clinicians.^2–5^

Bariatric surgery is an example of a treatment not being fully embraced by insurers despite RCT evidence of its significant benefits in terms of improved survival, sustained weight loss, and improvement in chronic conditions.^6,7^ Two seminal RCTs conducted in 2007 to 2011^8^ and 2009 to 2011^9^ demonstrated that the bariatric operations Roux-en-Y gastric bypass (RYGB) and sleeve gastrectomy were more effective than usual care for treating type 2 diabetes,^8,9^ an effect that was long lasting for most patients.^10,11^ These trials provided high-quality evidence supporting the use of bariatric surgery to treat obesity-related diabetes. However, the trials examined outcomes for a very selective population of patients and how these generalize to the overall population of patients undergoing bariatric surgery is unknown. Although outcomes for noneligible patients may be assumed from observational studies, these tend to overestimate beneficial effects because of poor follow-up of patients, biasing outcomes to those with better results who are more likely to follow-up after bariatric surgery.^12^

To overcome the limitations of the extant bariatric literature, we examined outcomes for patients undergoing bariatric surgery at University of California Los Angeles (UCLA) over a 5-year time period (2018–2023), comparing outcomes of patients eligible for trial enrollment with those who would not have been eligible. We sought to determine what proportion of patients who actually underwent surgery would have been eligible for enrollment in the RCTs and how outcomes for those who would not have been eligible compare to RCT results. By examining one population of patients, patterns of loss to follow-up would be similar in the groups compared, yielding more reliable estimates of how the RCT results compare to non-RCT eligible patient populations.

We hypothesized that (1) patients enrolled in the RCTs represent only a small proportion of patients undergoing bariatric surgery and (2) sleeve gastrectomy yields similar weight loss and diabetes control for patients who do and do not meet eligibility criteria for enrollment in surgical treatment and medications potentially eradicate diabetes efficiently (STAMPEDE) or diabetes surgery study (DSS).^8,9^

## METHODS

### Patient Population

**FIGURE 1.**
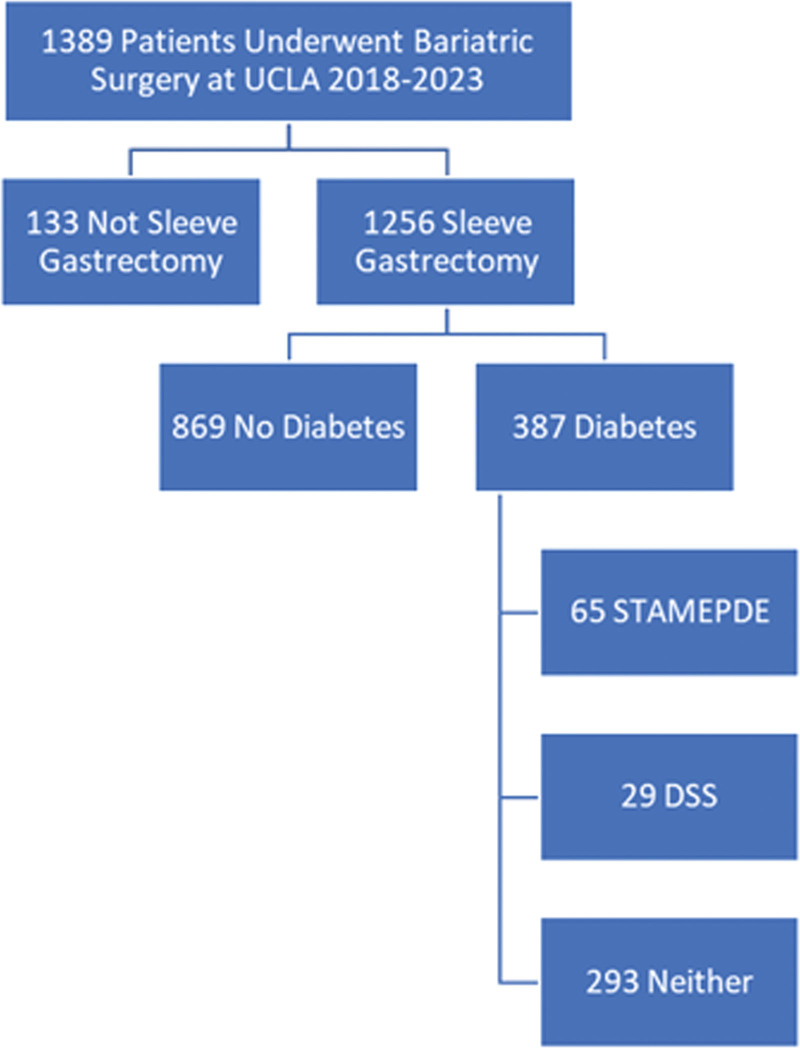
Flowchart of study patients. Of the 387 UCLA patients with diabetes, 29 fulfilled STAMPEDE inclusion criteria, 29 DSS, and 293 patients would not have been eligible for either study.

All patients with and without diabetes undergoing bariatric surgery between January /8, 2018 and May 19, 2023 were identified by current procedural terminology code 43774 for gastric sleeve gastrectomy (Fig. 1 and Supplemental Table 1, see http://links.lww.com/AOSO/A317). Patients undergoing gastric banding or revision procedures were excluded (n = 133). The final analytic samples included 1256 patients (387 with diabetes and 869 without diabetes). The study was approved by the UCLA Institutional Review Board (IRB# 22-001915).

### Clinical Data

Age, sex, race, and ethnicity were extracted from the UCLA Epic Clarity EHR database that includes all encounters at UCLA, after removal of restricted patients such as celebrities. Each patient encounter encoded for a diagnosis of diabetes, body mass index (BMI), serum glucose, and HbA1c in the 6 months before surgery was noted. Preoperative BMI was established by selecting the BMI measurement in the electronic medical record (EMR) closest to the date of surgery within 6 months before gastric sleeve gastrectomy (CRAN R module near date). Every measure of BMI and HbA1c available up to 5 years after the surgery was also obtained. One-year outcomes were assigned for any one measurement per patient available in the EMR between 6 and 18 months after surgery. Five-year data was similarly assigned for any data in the EMR between 54 and 66 months after surgery. When more than one measurement was available, a single value was randomly selected. BMI values in the medical records were excluded if they were <15 kg/m^2^ or >100 kg/m^2^. HbA1c values >15% were also excluded from the analysis.

UCLA patients were divided into 3 groups: Those fulfilling STAMPEDE entry criteria (BMI = 27–43 kg/m^2^; age, 20–60 years, and HbA1c > 7%), DSS (BMI = 30.0–39.9 kg/m^2^; age, 30–67 years; and HbA1c > 8.0%), or neither study.

Success of diabetes control after surgery was defined as having a postoperative HbA1c level of less than or equal to 7.0%.^13,14^

### Statistical Analysis

Cohorts were assembled from the UCLA patient sample who fulfilled, or did not fulfill, the study entry criteria for STAMPEDE and DSS. Baseline characteristics of the UCLA patients were compared with those of the RCTs’ patients calculating standardized mean differences (SMD) of means and percentages. Weight loss and HbA1c outcomes were also assessed by SMDs following sleeve gastrectomy and stratified by the presence or absence of a clinical diagnosis of diabetes. SMDs were calculated using an online calculator (https://www.campbellcollaboration.org/escalc/html/EffectSizeCalculator-SMD1.php) or directly in R. By convention, small differences (eg, small effect sizes) have SMD ≤ 0.5, medium 0.5–0.8, and the differences or effects considered large when the SMD > 0.8.^15^ In some instances, statistical significance between groups is presented with statistical significance defined as *P* < 0.05.

There were 2 analyses done just within the UCLA cohort.

First, heterogeneity in within-person change over time in BMI and HbA1c was explored with multilevel regression modeling. Time after surgery was treated as a random effect to account for repeated measures in our data. Because BMI and HbA1c responses were nonlinear, time was entered into the regression as a beta-spline. Treating time in this manner resulted in better regression fits than as a linear or quadratic function as tested by examining the smallest value for the Akaike information criterion. Age, Hispanic ethnicity, diabetes diagnosis, and study eligibility type were treated as fixed effects. Statistical significance of fixed effects in these models was determined by the Kenward–Roger adjusted *F* test. Second, baseline characteristics, weight change, and HbA1c change were compared between those who did or did not fulfill entry criteria for either STAMPEDE or DSS.

All statistical analyses were performed with the CRAN R program version 4.0.5.

## RESULTS

Of the 1256 patients who underwent sleeve gastrectomy in the analytic sample, 387 (31%) had a clinical diagnosis of diabetes. Baseline characteristics for these patients stratified by those eligible for STAMPEDE, DSS, and neither study are presented in Table 1 and Supplemental Table 2, see http://links.lww.com/AOSO/A317. Baseline characteristics for those UCLA patients who did or did not have diabetes are presented in Supplemental Table 3, see http://links.lww.com/AOSO/A317. The observed and modeled weight loss trajectory for all patients in the study is shown in Supplemental Figure 1, see http://links.lww.com/AOSO/A317. Patients with diabetes were more likely to be male than patients without diabetes, were older, and had a higher HbA1c. BMI, race, and ethnicity distribution were about the same between the groups. Despite not having a diagnostic code for diabetes, 12% of patients classified as not having diabetes had received prescriptions for insulin. Patients with diabetes who fulfilled STAMPEDE or DSS entry criteria had lower BMI, higher HbA1c levels, and were more likely to use insulin than those UCLA patients who would not have met these studies’ entry criteria. Comparison of baseline characteristics for UCLA patients who would have been eligible for STAMPEDE or DSS with those reported in the original articles is shown in Supplemental Table 4, see http://links.lww.com/AOSO/A317. HbA1c levels were higher in the STAMPEDE and DSS studies than in the UCLA cohort, but insulin usage was much higher in the UCLA patients, suggesting more aggressive diabetes control in the modern era.

**TABLE 1.** Baseline Characteristics for the UCLA-Sleeve Gastrectomy Cohort Who Have Diabetes and Do not Fulfill the Study Entry Criteria for STAMPEDE or DSS

	Neither	STAMPEDE	SMD*	DSS	SMD†
	(n = 318)	(n = 65)		(n = 30)	
Sex (% male)	97 (31%)	16 (25%)	0.13	7 (23%)	0.16
Age (yr)					
Mean (SD)	52 (±13)	49 (±7.7)	0.32	51 (±8.1)	0.08
BMI (kg//m^2^)					
Mean (SD)	44 (±7.1)	38 (±3.1)	1.1	36 (±2.3)	1.5
HbA1c (%)					
Mean (SD)	6.6 (±0.91)	7.5 (±1.4)	0.71	7.9 (±1.8)	0.94
Missing	35 (11.0%)	0 (0%)		0 (0%)	
Race					
American Indian or Alaska Native	6 (1.9%)	1 (1.5%)	0.027	1 (3.3%)	0.091
Asian	8 (2.5%)	8 (12.3%)	0.38	7 (23.3%)	0.653
Black or African American	51 (16.0%)	6 (9.2%)	0.206	3 (10.0%)	0.18
Middle Eastern or North African	8 (2.5%)	0 (0%)	0.227	1 (3.3%)	0.139
Native Hawaiian or Other Pacific Islander	4 (1.3%)	1 (1.5%)	0.024	9 (30.0%)	0.261
Other	135 (42.5%)	29 (44.6%)	0.044	9 (30.0%)	0.072
White or Caucasian	106 (33.3%)	20 (30.8%)	0.055	0 (0%)	0.227
Hispanic (%)	91 (29%)	25 (38%)	0.21	11 (37%)	0.17
Insulin (% prescribed)	197 (62%)	59 (91%)	0.72	27 (90%)	0.69

* STAMPEDE compared with neither.

† DSS compared with neither.

### STAMPEDE Analysis

Only 65 of the 387 (17%) patients with diabetes in our study fulfilled the STAMPEDE entry criteria. STAMPEDE ineligibility occurred in 164 (42%) of UCLA patients with diabetes because they had BMI > 43, 100 (26%) because they were older than 60 years, and 224 (58%) because their HbA1c was <7%. UCLA patients who would have been eligible for STAMPEDE had similar baseline characteristics to those UCLA patients not eligible for either study apart from lower HbA1c levels (9.5% ± 1.7% in STAMPEDE vs 7.9% ± 0.2% in the UCLA population measured 0–3 months before surgery).

HbA1c levels of UCLA patients measured between 3 and 6 months before surgery were considerably higher (8.3% ± 0.4%) than 0 to 3 months before surgery, consistent with preoperative medical and diabetes optimization. At 1-year after surgery, there was more weight loss in the STAMPEDE population relative to UCLA (1-year BMI, 27.2 ± 3.5 kg/m^2^ vs 31.0 ± 2.2 kg/m^2^; SMD = −1.26) but equivalent glucose control (HbA1c, 6.6% ± 1.0% vs 6.5% ± 1.3%; SMD = 0.09). In the original STAMPEDE study, there was some deterioration in glucose control manifested by a rise in HbA1c at 5 years (Table 2). Although there was only data from 5 patients in the UCLA cohort, weight loss was preserved but diabetes control was not as good as it was at 1 year. UCLA patients had better 1-year diabetes control than STAMPEDE with 50% having HbA1c ≤ 6% compared with 37% of sleeve gastrectomy patients in STAMPEDE.

**TABLE 2.** Comparison of Unadjusted BMI and Diabetes Control in UCLA Patients Fulfilling STAMPEDE Entry Criteria to Original STAMPEDE Results

	Stampede	STAMPEDE-RYGB			STAMPEDE-Sleeve			UCLA-Sleeve		
	Entry Criteria	Baseline	1 Yr	5 Yr	Baseline	1 Yr	5 Yr	Baseline	1 Yr	5 Yr
BMI	27–43	37.0 ± 3.3	26.8 ± 3.2	NR	36.2 ± 3.9	27.2 ± 3.5	NR	38.2 ± 0.4	31.0 ± 2.2	32.9 ± 2.9
Age	20–60	48.3 ± 8.4			47.9 ± 8.0			48.5 ± 1.0		
HbA1c	>7%	9.3 ± 1.4	6.4 ± 0.9	7.3 ± 1.5	9.5 ± 1.7	6.6 ± 1.0	7.4 ± 1.6	7.9 ± 0.2	6.5 ± 1.3	7.9 ± 2.2
n (patients)		50	50	47	50	49		62	20	5
% HbA1c ≤ 6.0%			21/50 (42%)	11/47 (23%)		18/49 (37%)			10/20 (50%)	1/5 (20%)

STAMPEDE results were those reported in the STAMPEDE publications.^8,11^ The column on the right represents data from UCLA patients who would have been eligible for STAMPEDE. HbA1c ≤ 6% was the criteria for diabetes treatment success used in STAMPEDE. The SMD between STAMPEDE-sleeve and UCLA 1-year BMI results is −1.26 and is 0.09 for HbA1c.

NR indicates not reported.

### DSS Analysis

Of the 387 UCLA patients with diabetes, 29 (7.5%) would have met the entry criteria for the DSS RCT (Table 3). UCLA patients would not have been eligible for DSS because 244 (63%) had BMI > 39.9, 13 (3%) were younger than 30 years, 40 (10%) were older than 67 years, and 194 (50%) had HbA1c < 8%. Of the 6 patients for whom 1-year follow-up data were available, substantial weight loss occurred (BMI, 36.0 ± 0.4 kg/m^2^ preoperatively vs 29.2 ± 1.2 4 kg/m^2^ 1 year after surgery). Diabetes control was excellent: the preoperative HbA1c of 8.3% ± 0.4% fell to 6.0% ± 1.2% after a year. The 1-year BMI for DSS was 25.8 ± 3.5 kg/m^2^, which was less than that observed for equivalent UCLA patients (29.2 ± 1.2, SMD = −1.15). The difference in HbA1c levels between the 2 groups at 1-year was small: 6.3% ± 0.9% versus 6.0% ± 1.2%, SMD = −0.30.

**TABLE 3.** Comparison of Unadjusted BMI and Diabetes Control in UCLA Patients Fulfilling DSS Entry Criteria and Original DSS Results

	DSS	DSS-RYGB			UCLA-Sleeve (n = 29)		
	Entry Criteria	Baseline	1 Yr	5 Yr	Baseline	1 Yr	5 Yr
BMI	30.0–39.9	34.9 ± 3.0	25.8 ± 3.5	27.4 ± 3.2	36.0 ± 0.4	29.2 ± 1.2	
Age	30–67	49 ± 9.0			51.1 + 1.5		
HbA1c	>8.0%	9.6 ± 1.0	6.3 ± 0.9	7.1 ± 1.5	8.3 + 0.4	6.0 ± 1.2	6.4
n (on insulin)		60	60	55	29	6	1
% HbA1c < 7.0%				31/55 (56%)		5/7 (71%)	

HbA1c ≤ 7% is the standard target for diabetes treatment. The SMD between STAMPEDE-Sleeve and UCLA 1-year BMI results is −1.15 and is 0.30 for HbA1c.

### Patients not Eligible for STAMPEDE or DSS

Most patients with diabetes (313/387, 81%) in our cohort would not have met the eligibility criteria for either RCT. These patients had higher baseline BMI (43.5 ± 8.1 kg/m^2^ vs 37.0 ± 3.3 kg/m^2^ for STAMPEDE and 34.9 ± 3.0 kg/m^2^ for DSS) and were older (51.6 ± 12.2 years vs 48.3 ± 8 years for STAMPEDE vs 49 ± 9 years for DSS) than those who enrolled in the RCTs but, on average, had lower HbA1c (7.0% ± 1.4% vs 9.3% ± 1.4% for STAMPEDE vs 9.6% ± 1.0% for DSS) (Table 4). Most patients had reasonable diabetes control before surgery with 61% having preoperative HbA1c < 7.0%. Weight loss following surgery was excellent (baseline BMI, 43.5 ± 8.1 kg/m^2^, fell to 34.7 ± 6.0 kg/m^2^ at 1 year and was 36.0 ± 7.8 kg/m^2^ at 5 years) and, for most patients, sustained (Table 4 and Supplemental Figure 1, see http://links.lww.com/AOSO/A317). Long-term diabetes control was similar to that observed in the RCTs results with average HbA1c levels for most patients being <7.0% at 1 (87%) and 5 (81%) years.

**TABLE 4.** UCLA Patients With diabetes not Fulfilling Either STAMPEDE or DSS Enrollment Criteria

	UCLA-Sleeve not STAMPEDE or DSS		
	Baseline	1 Yr	5 Yr
BMI	43.5 ± 8.1	34.7 ± 6.0	36.0 ± 7.8
Age	51.6 ± 12.2		
HbA1c	7.0 ± 1.4	6.1 ± 1.0	6.4 ± 1.1
n (BMI)	382	274	52
n (HbA1c)	307	208	67
% HbA1c < 7.0%	188/307 (61%)	199/208 (87%)	54/67(81%)

### Statistical Modeling of Weight Loss Over Time and the Effect of Study Eligibility

**FIGURE 2.**
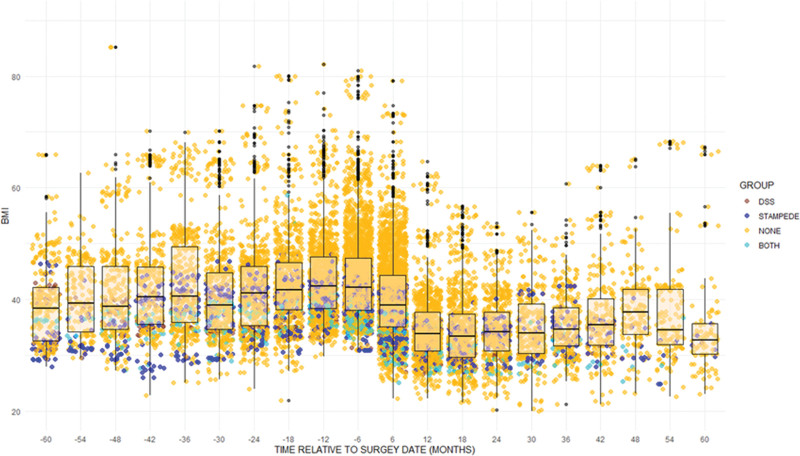
BMI of UCLA patients. Those who would have fulfilled STAMPEDE entry criteria are represented by blue dots, DSS, brown, and if they would have been eligible for both studies, turquoise. Orange dots represent BMI measurements for UCLA patients not meeting either study entry criterion.

**FIGURE 3.**
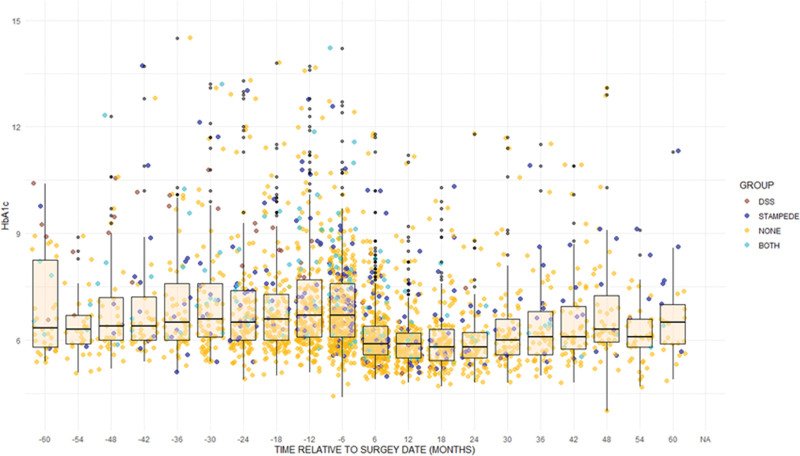
HbA1c UCLA patients. Those who would have fulfilled STAMPEDE entry criteria are represented by blue dots, DSS, brown, and if they would have been eligible for both studies, turquoise. Orange dots represent BMI measurements for UCLA patients not meeting either study entry criterion.

An analysis of weight change and HbA1c change in the UCLA cohort demonstrated an approximately 20% to 30% sustained weight loss and improved HbA1c for about 2 years with slight weight gain and loss of diabetes control thereafter (Figs. 2 and 3 and Supplemental Figures 6 and 7, see http://links.lww.com/AOSO/A317), with considerable heterogeneity. The scattergram shows that for bariatric populations, summary measures such as median values with interquartile ranges poorly represent the total population as many measures are beyond the 25th and 75th percentiles, and there are many outliers. In general, patients fulfilling entry criteria for the RCTs were at the lower end of BMI and higher end of HbA1c measurements. The scatterplots show that, in general, there is a general trend of improvement following weight loss surgery irrespective of baseline levels of obesity or glucose control.

In the second analysis comparing outcome changes between those who were and were not eligible for the 2 major RCTs, BMI was significantly different (Kenward–Roger adjusted *F* test, *P* = 0) between STAMPEDE eligible and ineligible patients in the UCLA cohort, with BMI consistently higher for ineligible patients (Supplemental Figure 2, see http://links.lww.com/AOSO/A317). Age and Hispanic ethnicity did not significantly affect the BMI versus time curve. Sex had a statistically significant effect, but it was very small. Being not eligible or eligible for STAMPEDE did result in statistically significant differences and had a reasonably large effect size (Supplemental Figure 3, see http://links.lww.com/AOSO/A317). A similar BMI comparison between DSS eligible and ineligible patients in the UCLA cohort showed that BMI change was similar (Supplemental Figure 4, see http://links.lww.com/AOSO/A317). In general, even though the ineligible patients had higher BMIs, the modeled BMI trajectories were roughly parallel.

HbA1c responses were more erratic and inconsistent than those for BMI (Supplemental Figure 5 and 6, see http://links.lww.com/AOSO/A317). STAMPEDE eligible patients had higher HbA1c levels throughout the study (*P* = 0.0097) than ineligible patients in the UCLA cohort, with different trajectories (Supplemental Figure 7, see http://links.lww.com/AOSO/A317). Similar analyses were not performed for DSS eligible patients because of the small number of patients, precluding regression analysis.

## DISCUSSION

Most patients undergoing bariatric surgery in a large West Coast academic medical center differed substantially from those enrolled in the pivotal trials demonstrating the superiority of bariatric surgery over medical treatment for diabetes. In fact, only a small proportion of diabetic patients in our study fulfilled the entry criteria for STAMPEDE or DSS primarily from having too low of HbA1c levels, having greater BMIs, or were older. Differences between baseline patient characteristics treated in real-world practice and those studied in RCTs raise potential concerns about the generalizability of RCT outcomes to the universe of patients undergoing treatment in everyday clinical practice if those differences result in different outcomes.^1,16,17^ However, our study showed that patients who were very different from those in the RCTs achieved similar weight loss and diabetes control, which suggests that outcomes from STAMPEDE and DSS generalize to most patients with diabetes currently undergoing bariatric surgery.

On average, the UCLA cohort had slightly higher BMIs than the RCT population and lower preoperative HbA1c levels. Time trend analyses of the UCLA cohort showed that HbA1c levels 6 to 12 months before surgery were higher than 0 to 6 months before surgery, consistent with preoperative diabetes optimization embedded in contemporary bariatric surgery programs. Nonetheless, postoperative diabetes control in the UCLA population mimicked that from the RCTs. RCTs present outcomes for ideal patients receiving treatment under optimal circumstances. RCT outcomes represent an upper bound for a range of possible outcomes for any intervention because resources available in RCTs are generally more than those available for routine clinical care. That UCLA patients had similar diabetes control outcomes to those of the RCTs, which implies that sleeve gastrectomy results for diabetes control generalize well beyond the RCTs for equivalent populations.

It is more the rule than the exception that RCTs enroll unique subpopulations of the universe of patients in need of the treatments being investigated by the RCTs. Examples include treatment for hypertension,^18^ cognitive impairment,^19^ bipolar disorder,^20^ HIV,^21,22^ mouth disorders,^23^ and many other disease states.^24^ To incorporate RCT findings into clinical practice recommendations, it is essential that their results be assessed for generalizability. This is not frequently done. However, the advent of EMRs with large databases accumulating outcomes data for routine clinical care and availability of transportability methods^25–27^ can facilitate the sort of analysis we performed for diabetes control by bariatric surgery. Before applying RCT findings into routine care, clinicians should first know if their patients have the same characteristics as those enrolled in the RCT. If they differ from those studied in RCTs, then clinicians should rely on data showing that these patients will have the same outcomes as predicted by the RCT. For this to occur, observational data analysis, as was performed in the current study, is necessary to inform clinical decision-making.

Statistical modeling is sometimes used to predict outcomes for patients who would not meet enrollment criteria in an RCT.^28,29^ A major limitation of this approach is that prediction models are only valid within the bounds of the covariates entered into the model.^29,30^ Extrapolation of predictions derived from covariates beyond the bounds of those used to develop the model may yield misleading results, but work in this area is ongoing. For our study, models predicting diabetes outcomes for patients with BMI exceeded those enrolled in STAMPEDE or DSS. As seen in Figure 1, many patients with diabetes undergoing bariatric surgery had BMIs much higher than those of the STAMPEDE or DSS populations.

Summary statistical results of our study are like the many others of bariatric surgery outcomes demonstrating that for a population of patients, on average, there is sustained weight loss and HbA1c reduction following surgery.

All the patients in the UCLA cohort underwent sleeve gastrectomy. Whereas in the past they were the dominant operation,^31^ very few RYGBs are now performed in our practice. Patients in STAMPEDE were equally divided into groups that had RYGB or sleeve gastrectomy procedures. All patients in DSS had RYGBs. Conceivably, our findings may not generalize to RYGB procedures. Observational studies investigating diabetes outcomes for RYGB and sleeve gastrectomy found slightly better weight loss and diabetes outcomes for RYGB when compared with sleeve gastrectomy.^13,32^ However, RCTs comparing the 2 procedures found diabetes control was equivalent for the 2 procedures.^33–35^ Diabetes control outcomes between the procedures also depend on diabetes severity. Diabetes control was equivalent between RYGB and sleeve gastrectomy for patients with severe and mild diabetes whereas intermediate diabetes severity was associated with better outcomes from RYGB.^36^ We cannot predict outcomes from RYGB based on our analysis for patients with characteristics that differ from those of the RCTs. However, in general, diabetes control outcomes in our study were excellent for a wide range of patients resulting from the much simpler sleeve gastrectomy procedure.

Our study has limitations. First, our data were a sample of data available with the UCLA health system. We did not have access to all BMI and HbA1c data for patients receiving part of their health care outside of our system, which was partially offset by having large amounts of detailed information about UCLA Health patients obtained over the time that was available for follow-up that we could assess. Second, follow-up for our patients was not systematic but occurred as patients’ needs arose, which resulted in too few patients to enable more extensive comparisons with the DSS trial that had several, simultaneous outcomes measured. Even though only a very small proportion of patients undergoing bariatric surgery would have been eligible for entry into the major RCTs, the overall population had similar outcomes, suggesting that diabetes outcomes from STAMPEDE and DSS generalize to most patients undergoing bariatric surgery for diabetes control. These data were from a single health system and may not generalize to other care environments or geographic locations. Because our data were derived from clinic encounters and many patients (~30% to 40%) did not report it, we are uncertain how race or ethnicity might influence our results. Third, treatment success was defined differently in the STAMPEDE and DSS trials and our study. STAMPEDE defined success as no longer having diabetes with the definition of having diabetes as having HbA1c > 6.0%. DSS’s definition of success was achieving the American Diabetes Association triple point endpoint: composite goal of HbA1c less than 7.0%, low-density lipoprotein cholesterol less than 100 mg/dL, and systolic blood pressure less than 130 mm Hg. We defined success as having an HbA1c < 7.0%, which is the American Diabetes Association’s treatment goal for diabetes in terms of glucose control alone. Each of these are legitimate treatment goals and results of our study placed in the context of these differing goals.

## Supplementary Material


