# Supplementary material for: Generalizability of Randomized Clinical Trial Outcomes for Diabetes Control Resulting From Bariatric Surgery
Source: Ann Surg Open. 2024 Apr 10;5(2):e414. doi: 10.1097/AS9.0000000000000414 (PMC11192007; doi:10.1097/AS9.0000000000000414)
Supplement: Supplementary file 1 [file as9-5-e414-s001.pdf]

SUPPLEMENT

sTable 1. CPT Codes used to identify UCLA bariatric procedures from 1/1/2017 to 5/19/2023. Only procedures encoded with CPT=43775 were included in this study.

CPT	n	Description
42771	1	Lap, revise adjustable gastric restrictive device
42772	3	Lap, remove adjustable gastric restrictive device
42773	1	Lap, change adjustable gastric restrictive device
42774	128	Lap,remove adjustable gastric restrictive device
42775	1256	Lap, gastric restrictive procedure, longitudinal gastrectomy
Total	1389	

sTable 2. Baseline Characteristics for the Patients Included in This Analysis with P values.

	Neither (N=318)	STAMPEDE (N=65)	P-value[1]	DSS (N=30)	P-value[2]
**Sex**					
Female	221 (69 %)	49 (75 %)	0.424	23 (77 %)	0.541
Male	97 (31 %)	16 (25 %)		7 (23 %)	
**Age (Years)**					
Mean (SD)	52 (± 13)	49 (± 7.7)	0.00494	51 (± 8.1)	0.599
**BMI (kg/m2)**					
Mean (SD)	44 (± 7.1)	38 (± 3.1)	<0.001	36 (± 2.3)	<0.001
**A1c (%)**					
Mean (SD)	6.6 (± 0.91)	7.5 (± 1.4)	<0.001	7.9 (± 1.8)	<0.001
Missing	35 (11.0%)	0 (0%)		0 (0%)	
**Race**					
American Indian or Alaska Native	6 (2 %)	1 (2 %)	0.0141	1 (3 %)	<0.001
Asian	8 (3 %)	8 (12 %)		7 (23 %)	
Black or African American	51 (16 %)	6 (9 %)		3 (10 %)	
Middle Eastern or North African	8 (3 %)	0 (0 %)		1 (3 %)	
Native Hawaiian or Other Pacific Islander	4 (1 %)	1 (2 %)		9 (30 %)	
Other	135 (42 %)	29 (45 %)		9 (30 %)	
White or Caucasian	106 (33 %)	20 (31 %)		0 (0 %)	
**Hispanic**					
Not Hispanic	227 (71 %)	40 (62 %)	0.154	19 (63 %)	0.474
Hispanic	91 (29 %)	25 (38 %)		11 (37 %)	
**Insulin**					
No Insulin	121 (38 %)	6 (9 %)	<0.001	3 (10 %)	0.00414
Uses Insulin	197 (62 %)	59 (91 %)		27 (90 %)	

1-STAMPEDE compared with Neither

2-DSS compared with Neither

sTable 3. Baseline Characteristics for UCLA SG Patients Who Had or Did Not Have DM

UCLA Sleeve Resection Patients 2018-2023			
	**No Diabetes (N=869)**	**Diabetes (N=387)**	**P-value**
**Sex**			
Female	694 (80 %)	272 (70 %)	<0.001
Male	174 (20 %)	115 (30 %)	
Missing	1 (0.1%)	0 (0%)	
**Age (Years)**			
Mean (SD)	44 (± 12)	52 (± 12)	<0.001
**BMI (kg/m2)**			
Mean (SD)	44 (± 6.1)	43 (± 7.0)	0.279
Missing	1 (0.1%)	0 (0%)	
**A1c (%)**			
Mean (SD)	5.7 (± 0.36)	6.8 (± 1.1)	<0.001
Missing	125 (14.4%)	35 (9.0%)	
**Race**			
American Indian or Alaska Native	8 (1 %)	7 (2 %)	0.0384
Asian	17 (2 %)	18 (5 %)	
Black or African American	112 (13 %)	58 (15 %)	
Middle Eastern or North African	22 (3 %)	8 (2 %)	
Native Hawaiian or Other Pacific Islander	7 (1 %)	5 (1 %)	
Other	364 (42 %)	164 (42 %)	
White or Caucasian	339 (39 %)	127 (33 %)	
**Hispanic**			
Not Hispanic	585 (67 %)	271 (70 %)	0.376
Hispanic	284 (33 %)	116 (30 %)	
**Insulin**			
No Insulin	765 (88 %)	127 (33 %)	<0.001
Uses Insulin	104 (12 %)	260 (67 %)	

sTable 4. Comparison of baseline characteristics of UCLA patients who would have been eligible for STAMPEDE or DSS and the baseline characteristics for patient enrolled in those studies as reported in the pinitol articles.

	STAMPEDE-UCLA (N=65)	STAMPEDE-Schauer[1] n=50	DSS-UCLA (N=30)	DSS (Ikramuddin)[2]
**SEX (% Male)**				
Mean (SD)	0.25 (± 0.43)	0.22	0.23 (± 0.43)	0.37
**AGE (Years)**				
Mean (SD)	49 (± 7.7)	47.9(±8.0)	51 (± 8.1)	49 (± 9)
**BMI (kg//m^2)**				
Mean (SD)	38 (± 3.1)	36.2±3.9	36 (± 2.3)	34.9 (± 3.0)
**A1c (%)**				
Mean (SD)	7.5 (± 1.4)	9.5 (± 1.7)	7.9 (± 1.8)	9.6 (± 1.0)
Missing	0 (0%)		0 (0%)	
**RACE**				
American Indian or Alaska Native	1 (1.5%)		1 (3.3%)	3%
Asian	8 (12.3%)		7 (23.3%)	27%
Black or African American	6 (9.2%)		3 (10.0%)	8%
Middle Eastern or North African	0 (0%)		1 (3.3%)	
Native Hawaiian or Other Pacific Islander	1 (1.5%)		9 (30.0%)	
Other	29 (44.6%)		9 (30.0%)	0%
White or Caucasian	20 (30.8%)		0 (0%)	55%
**HISPANIC (%)**				
Mean (SD)	0.38 (± 0.49)		0.37 (± 0.49)	0.04
**INSULIN (% Prescribed)**				
Mean (SD)	0.91 (± 0.29)	0.32	0.90 (± 0.31)	0.62

1-STAMPEDE eligible UCLA patients compared with sleeve patient reported in Schaeur (NEJM 2012)

2-UCLA patients eligible for DSS compared with RYGB pateints reported by Ikramuddin (JAMA 2013)

WEIGHT LOSS MODELS FOLLOWING BARIATRIC SURGERY

PATIENTS NOT FULFILLING ANY RCT CRITERIA v STAMPEDE

Optimize Time Variable

To determine the best fit between the BMI-time from surgery (in months) relationship, a series of regressions was performed, modeling time as a linear function, quadratic and beta-spline with 3 degrees of freedom. The regression with the smallest Akaike Information Criteria (AIC) was the one with time modeled as a beta-spline, consequently, beta-splines were applied to the time variable in all subsequent models. Of note, models using beta-splines with 4 degrees of freedom failed to converge in subsequent analyses.

```
BMIfit <- lm((BMI ~ BMI_MONTHS), data=BMIDM_Temp)   #AIC=33447

BMIfit <- lm(BMI ~ BMI_MONTHS + I(BMI_MONTHS^2), data=BMIDM_Temp)  #AIC=33147

BMIfit <- lm(BMI ~ bs(BMI_MONTHS,df=3), data=BMIDM_Temp)  #AIC=32887
```

Modeling BMI Outcomes

Stepwise development of the mixed model for changes in BMI with time after surgery. “Study” refers to patients either being eligible for STAMPEDE or neither STAMPEDE nor DSS. Time was treated as a random effect whereas study, age, Hispanic ethnicity and sex were all treated as fixed effects. The largest improvement in AIC was following the addition of study as a fixed effect to the model that had time as a random effect for both intercept and slope. Addition of age, sex and Hispanic ethnicity only minimally improved the regression model fit of the data.

```
BMIfit <- lmer(BMI ~ (bs(BMI_MONTHS,df=3) | IP_PATIENT_ID), data=BMIDM_Temp)  #AIC=21027
# Months random effect-slope only

BMIfit <- lmer(BMI ~ (1 +bs(BMI_MONTHS,df=3) | IP_PATIENT_ID), data=BMIDM_Temp) #AIC=21027
# Months random effect-slope and intercept

BMIfit <- lmer(BMI ~ Study + (1 +bs(BMI_MONTHS,df=3) | IP_PATIENT_ID), data=BMIDM_Temp)
#AIC=20991    # Months slope/intercetp RE _ Study
```

```
BMIfit <- lmer(BMI ~ Study + AGE + HISPANIC + SEX + (1 +bs(BMI_MONTHS,df=3) | IP_PATIENT_ID),
data=BMIDM_Temp) #AIC=20990
# Full model
```

sFigure 1. Individual postoperative BMI measurements and modeled data

```
preds <- predict(BMIfit, newdata=BMIDM_Temp)

ggplot(BMIDM_Temp, aes(BMI_MONTHS, preds, color = factor(IP_PATIENT_ID))) +    # plot individual
BMIs and spline fits per patient
  geom_line(show.legend=FALSE) +
  geom_point(data = BMIDM_Temp, aes(y = BMI)) +
  guides(color = guide_none()) +
  labs(y="Observed (Points) and Predicted (Lines) BMI",x="Months After Surgery" )
```

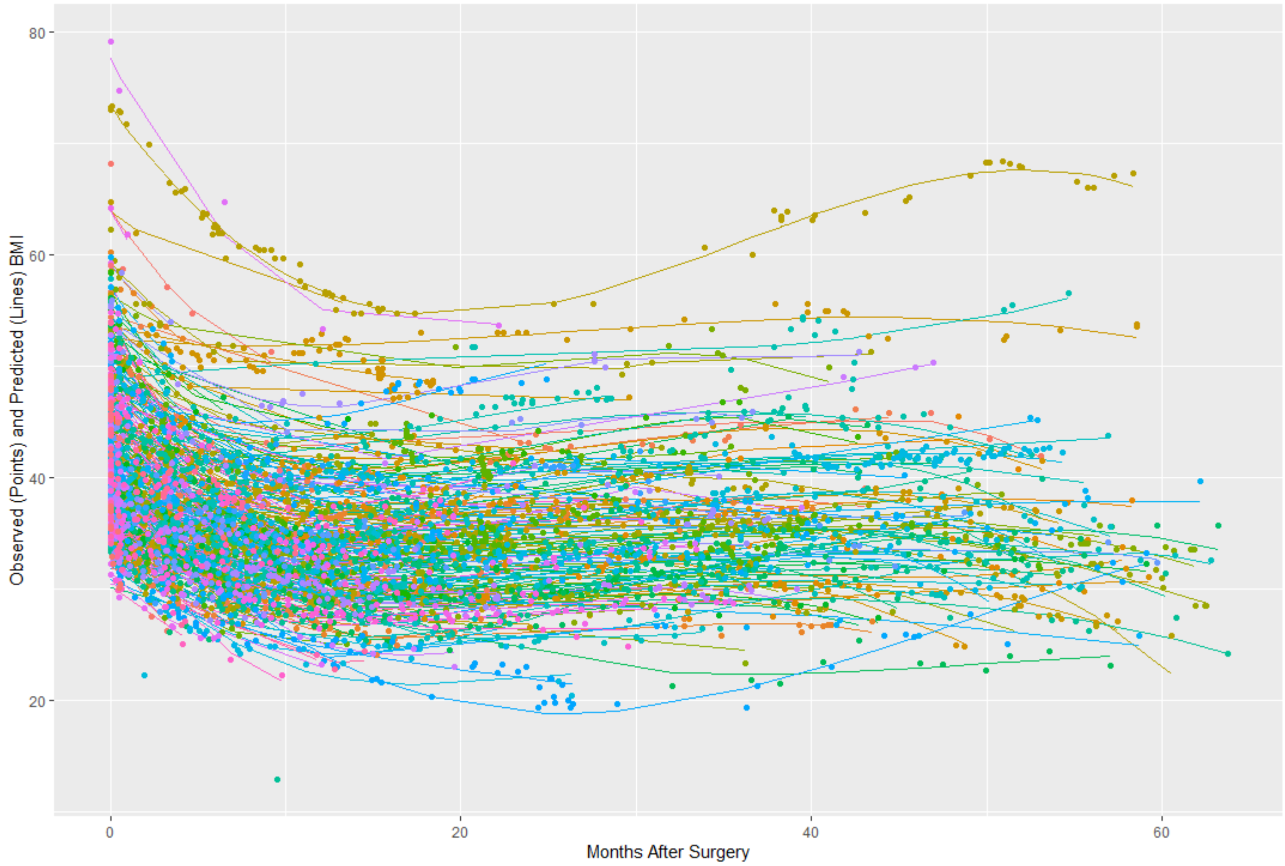

Solid circles represent individual BMI measurements for patients relative to the date when they underwent surgery (time=0). The curves are fitted BMI trajectories for each patient. The colors are the same for each patient's observed BMI measurement and the fitted prediction.

Full model-Ineligible v STAMPEDE (Full Model)

```
> summary(BMIfit)
Linear mixed model fit by REML. t-tests use Satterthwaite's method ['lmerModLmerTest']
Formula: BMI ~ Study + AGE + HISPANIC + SEX + (1 + bs(BMI_MONTHS, df = 3) |
    IP_PATIENT_ID)
   Data: BMIDM_Temp

REML criterion at convergence: 20958.4

Scaled residuals:
     Min       1Q   Median       3Q      Max
-15.6914  -0.4713  -0.0122   0.4502   5.8308

Random effects:
 Groups        Name                     Variance Std.Dev. Corr
 IP_PATIENT_ID (Intercept)                60.881  7.803
               bs(BMI_MONTHS, df = 3)1 1185.659 34.433   -0.65
               bs(BMI_MONTHS, df = 3)2 1871.268 43.258    0.50 -0.89
               bs(BMI_MONTHS, df = 3)3 5293.780 72.758   -0.50  0.91 -0.97
 Residual                                  1.814  1.347
Number of obs: 4894, groups:  IP_PATIENT_ID, 383

Fixed effects:
               Estimate Std. Error        df t value Pr(>|t|)
(Intercept)    42.36737    1.42705 374.52578  29.689  < 2e-16 ***
StudySTAMPEDE  -5.49893    0.82749 386.13641  -6.645 1.03e-10 ***
AGE            -0.07776    0.02559 373.93501  -3.039  0.00254 **
HISPANIC1       0.22659    0.69233 384.32101   0.327  0.74363
SEX1            0.47422    0.67530 377.62796   0.702  0.48296
---
Signif. codes:  0 '***' 0.001 '**' 0.01 '*' 0.05 '.' 0.1 ' ' 1

Correlation of Fixed Effects:
            (Intr) SSTAMP AGE    HISPAN
StdSTAMPEDE -0.170
AGE         -0.953  0.084
```

```
HISPANIC1   -0.339 -0.075  0.227
SEX1        -0.030  0.044 -0.116 -0.049 > anova(BMIfit)
Type III Analysis of Variance Table with Satterthwaite's method
         Sum Sq Mean Sq NumDF  DenDF F value    Pr(>F)    
Study    58.613  58.613     1 385.51 45.1053 6.712e-11 ***
AGE      10.738  10.738     1 373.69  8.2633  0.004277 ** 
HISPANIC  0.014   0.014     1 385.12  0.0105  0.918249    
SEX       0.301   0.301     1 378.02  0.2315  0.630723    
---
```

Kenward-Roger adjusted F-test for the effect of the STAMPEDE Study

```
BMIfitR <- lmer(BMI ~  AGE + HISPANIC + SEX + (1 +bs(BMI_MONTHS,df=3) | IP_PATIENT_ID), data=BMIDM_Temp, REML=FALSE)

BMIfit <- lmer(BMI ~  AGE + HISPANIC + SEX + Study + (1 +bs(BMI_MONTHS,df=3) | IP_PATIENT_ID), data=BMIDM_Temp, REML=FALSE)

KRmodcomp(BMIfit,BMIfitR)


large : BMI ~ AGE + HISPANIC + SEX + Study + (1 + bs(BMI_MONTHS, df = 3) | 

    IP_PATIENT_ID)

small : BMI ~ AGE + HISPANIC + SEX + (1 + bs(BMI_MONTHS, df = 3) | IP_PATIENT_ID)

         stat     ndf     ddf F.scaling   p.value    

Ftest  43.421   1.000 379.928         1 1.476e-10 ***

---

Signif. codes:  0 '***' 0.001 '**' 0.01 '*' 0.05 '.' 0.1 ' ' 1
```

Graphs

sFigure 2. Weight Loss After Bariatric Surgery-Stratify by No RCT Criteria v STAMPEDE

```
ggplot(BMIDM_Temp, aes(BMI_MONTHS, BMI) ) +   geom_point() +
  stat_smooth(data=subset(BMIDM_Temp,Study=='Neither'),color='green',method="lm",formula = y ~ bs(x,3)) +
  stat_smooth(data=subset(BMIDM_Temp,Study=='STAMPEDE'),color='red',method="lm",formula = y ~ bs(x,3))
```

Plot of individual BMI measurements (black dots) following bariatric surgery. The green line represents fitted data for patients not eligible for study entry into DSS or STAMPEDE and the redline is for those eligible for STAMPEDE. The regression displayed here is for fixed effects only, with no covariates and time modeled as a beta-spline with 3 df. In general, patients not eligible for STAMPEDE follow a roughly parallel trajectory to those who were eligible. The rate of weight loss was slightly less for the STAMPEDE group, consistent with the negative slope of the Study coefficient in the random effects regression (above).

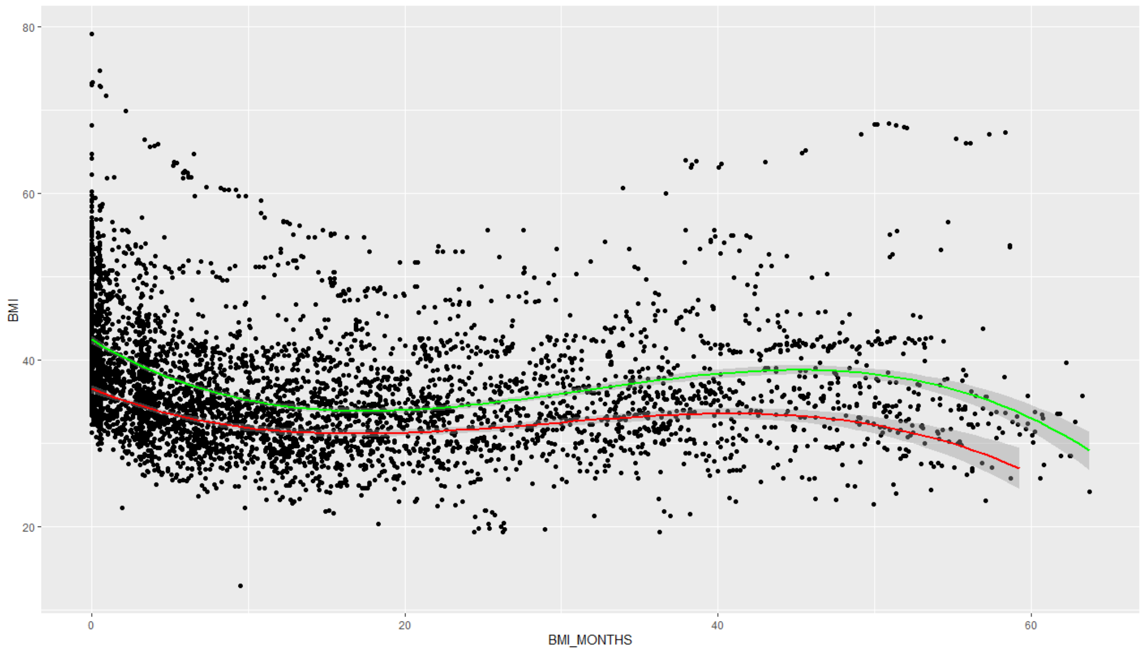

sFigure 3. Effect size of the fixed effects

```
sjPlot::plot_model(BMIfit,axis.labels=c("Sex", "Hispanic", "Age","None v STAMPEDE"),show.values=TRUE,
show.p=TRUE,title="BMI Model for STAMPEDE")
```

Age and Hispanic ethnicity did not significantly affect the BMI v time curve. Sex had a statistically significant effect, but it was very small. Being not eligible or eligible for STAMPEDE did result in statistically significant differences and had a reasonably large effect size.

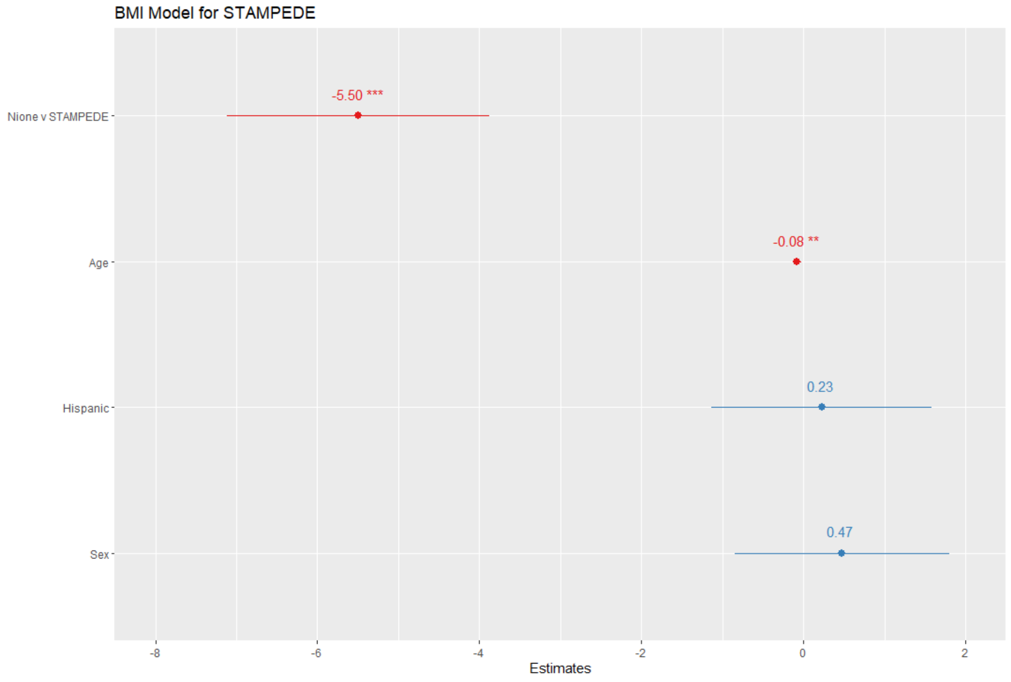

Full model-Ineligible v DSS (Full Model)

```
BMIfit <- lmer(BMI ~ Study + AGE + HISPANIC + SEX + (1 +bs(BMI_MONTHS,df=3) | IP_PATIENT_ID),
data=BMIDM_Temp)

summary(BMIfit)
Linear mixed model fit by REML. t-tests use Satterthwaite's method ['lmerModLmerTest']
Formula: BMI ~ Study + AGE + HISPANIC + SEX + (1 + bs(BMI_MONTHS, df = 3) |
    IP_PATIENT_ID)
   Data: BMIDM_Temp

REML criterion at convergence: 18207.3

Scaled residuals:
     Min       1Q   Median       3Q      Max
-15.5313  -0.4578  -0.0158   0.4365   5.7777

Random effects:
 Groups        Name                    Variance Std.Dev. Corr
 IP_PATIENT_ID (Intercept)               68.619  8.284
               bs(BMI_MONTHS, df = 3)1 1215.162 34.859   -0.65
               bs(BMI_MONTHS, df = 3)2 1711.085 41.365    0.49 -0.89
               bs(BMI_MONTHS, df = 3)3 4403.199 66.357   -0.49  0.91 -0.97
 Residual                                 1.852  1.361
Number of obs: 4238, groups:  IP_PATIENT_ID, 323

Fixed effects:
              Estimate Std. Error        df t value Pr(>|t|)
(Intercept)   38.41005    3.36094 320.92026  11.428  < 2e-16 ***
StudyNeither   4.00430    2.91515 322.61396   1.374  0.17051
AGE           -0.08290    0.02793 313.37491  -2.969  0.00322 **
HISPANIC1      0.52544    0.81632 325.30222   0.644  0.52025
SEX1           0.54733    0.77140 317.18457   0.710  0.47851
---
Signif. codes:  0 '***' 0.001 '**' 0.01 '*' 0.05 '.' 0.1 ' ' 1
```

```
Correlation of Fixed Effects:
            (Intr) StdyNt AGE    HISPAN
StudyNeithr -0.886                     
AGE         -0.512  0.081              
HISPANIC1   -0.102 -0.057  0.206       
SEX1        -0.023  0.006 -0.110 -0.067
```

sFigure 4. Weight Loss After Bariatric Surgery-Stratify by No RCT Criteria v DSS

```
ggplot(BMIDM_Temp, aes(BMI_MONTHS, BMI) ) +   geom_point() +
  stat_smooth(data=subset(BMIDM_Temp,Study=='Neither'),color='green',method="lm",formula = y ~ bs(x,3)) +
  stat_smooth(data=subset(BMIDM_Temp,Study=='DSS'),color='red',method="lm",formula = y ~ bs(x,3))
```

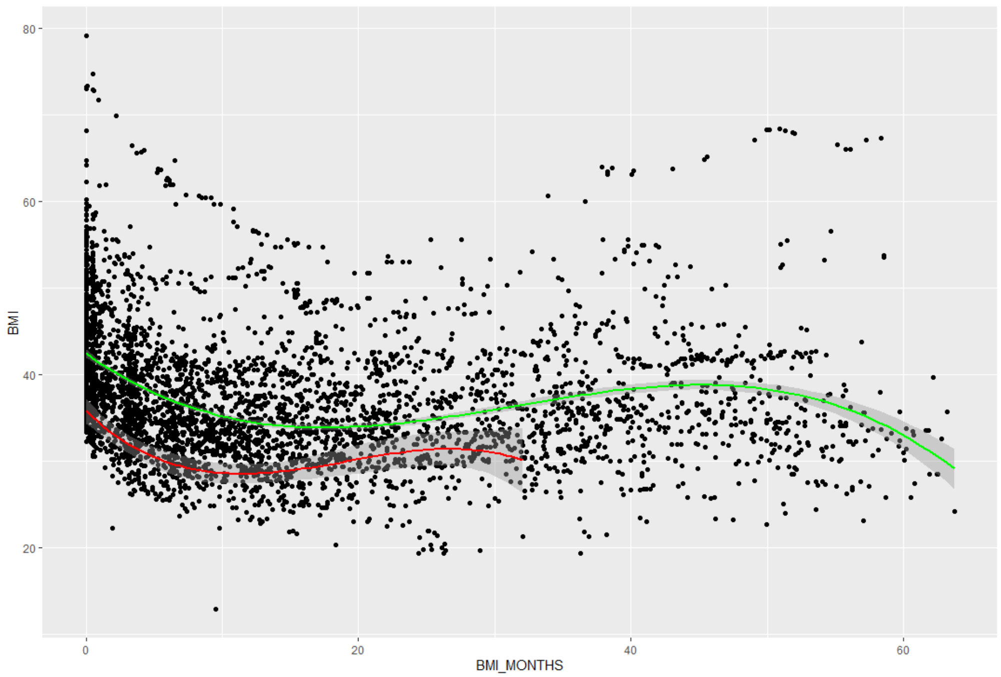

MODELS OF THE HbA1c RESPONSE TO WEIGHT LOSS FOLLOWING BARIATRIC SURGERY

sFigure 5. Visualization of the Relationships

```
ggplot(dfDM_Temp, aes(A1c_MONTHS, A1c, color = factor(IP_PATIENT_ID))) +
geom_line(show.legend=FALSE) +
geom_point(data = dfDM_Temp, aes(y = A1c),show.legend=FALSE)
```

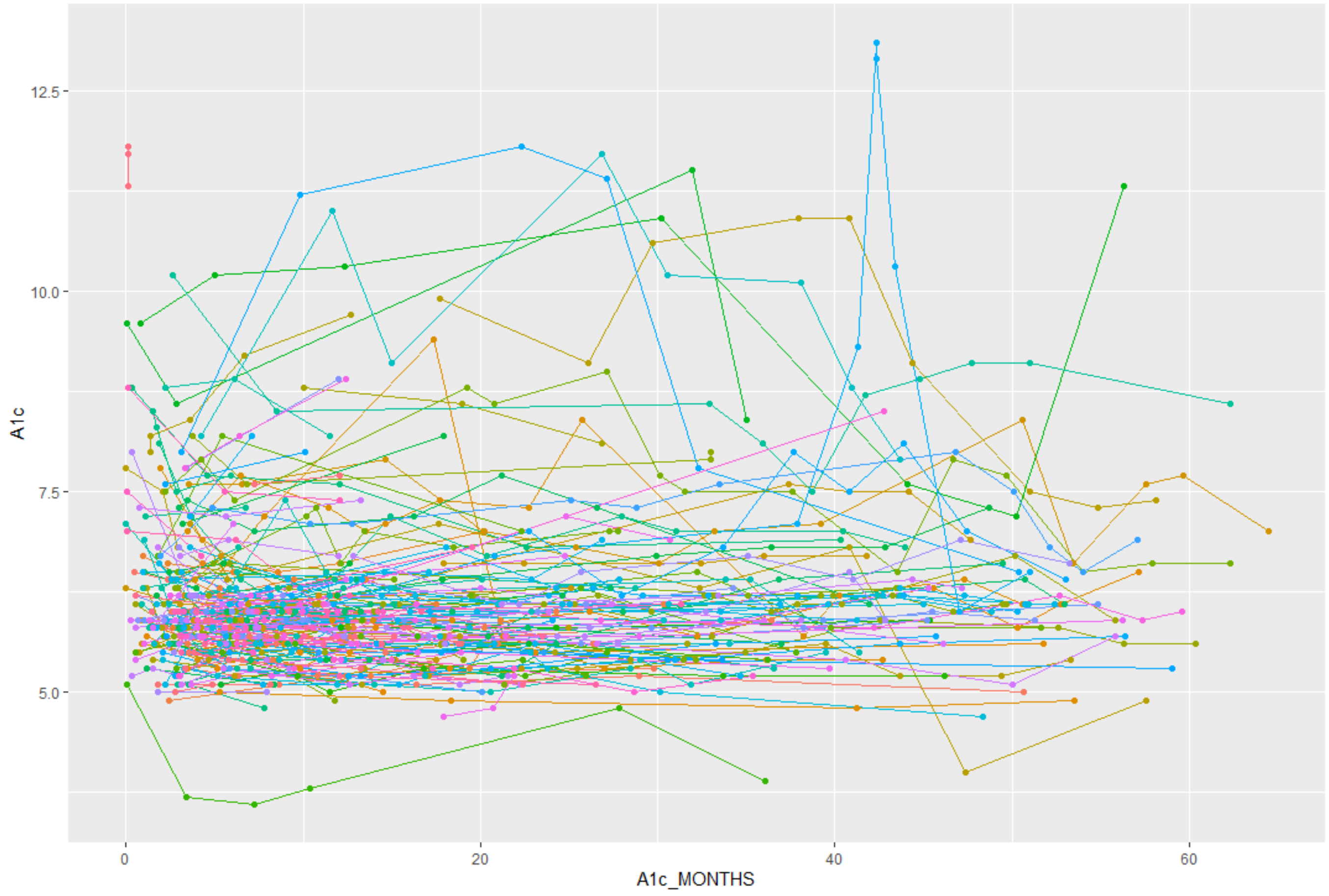

Spaghetti plot of observed measured A1c values over time following surgery. Most patients have A1c values in the normal range. For those with elevated A1c, there is a tendency to have a poor response to surgery.

AIC Analysis of Not Eligible v STAMPEDE Models

```
dffit <- lmer(A1c ~ (bs(A1c_MONTHS,df=3) | IP_PATIENT_ID), data=dfDM_Temp)  #AIC=2099
# Months random effect-slope only

dffit <- lmer(A1c ~ (1 +bs(A1c_MONTHS,df=3) | IP_PATIENT_ID), data=dfDM_Temp) #AIC=2099
# Months random effect-slope and intercept

 dffit <- lmer(A1c ~ Study*A1c_MONTHS + (1 +bs(A1c_MONTHS,df=3) | IP_PATIENT_ID), data=dfDM_Temp)
#AIC=2117 Interation between Study and Time

dffit <- lmer(A1c ~ Study + AGE + (1 +bs(A1c_MONTHS,df=3) | IP_PATIENT_ID), data=dfDM_Temp)
#AIC=2098

dffit <- lmer(A1c ~ Study + AGE + HISPANIC + SEX +(1 +bs(A1c_MONTHS,df=3) | IP_PATIENT_ID),
data=dfDM_Temp) #AIC=2101 Full Model
```

Addition of the study type (not eligible v STAMPEDE eligible) and age resulted in the best fit (eg, having the smallest AIC). The full model fits slightly worse. Time was modeled as a beta-spline with 3 degrees of freedom.

```
dffit <- lmer(A1c ~ Study + AGE + (1 +bs(A1c_MONTHS,df=3) | IP_PATIENT_ID), data=dfDM_Temp)

summary(dffit)

Linear mixed model fit by REML. t-tests use Satterthwaite's method ['lmerModLmerTest']
Formula: A1c ~ Study + AGE + (1 + bs(A1c_MONTHS, df = 3) | IP_PATIENT_ID)
   Data: dfDM_Temp

REML criterion at convergence: 2070.2
```

```
Scaled residuals:
    Min      1Q  Median      3Q     Max 
-5.7432 -0.2805 -0.0511  0.2127  7.3628 

Random effects:
 Groups        Name                         Variance Std.Dev. Corr             
 IP_PATIENT_ID (Intercept)                   0.8721  0.9339                    
               bs(A1c_MONTHS, df = 3)1  6.8325  2.6139   -0.30             
               bs(A1c_MONTHS, df = 3)2 11.2678  3.3568    0.37 -0.84       
               bs(A1c_MONTHS, df = 3)3  6.7290  2.5940   -0.53  0.76 -0.95
 Residual                                    0.2263  0.4757                    
Number of obs: 933, groups:  IP_PATIENT_ID, 215

Fixed effects:
               Estimate Std. Error        df t value Pr(>|t|)    
(Intercept)   5.346e+00  2.829e-01 2.141e+02  18.894  < 2e-16 ***
StudySTAMPEDE 5.693e-01  1.760e-01 2.180e+02   3.234  0.00141 ** 
AGE           1.360e-02  5.045e-03 2.074e+02   2.696  0.00760 ** 
---
Signif. codes:  0 '***' 0.001 '**' 0.01 '*' 0.05 '.' 0.1 ' ' 1

Correlation of Fixed Effects:
            (Intr) SSTAMP
StdSTAMPEDE -0.182       
AGE         -0.970  0.088
```

Test statistical significance of the fixed effects age and Study group with the Kenward-Roger approximation

Testing for the statistical significance of age.

```
dffitR <- lmer(A1c ~ Study + (1 +bs(A1c_MONTHS,df=3) | IP_PATIENT_ID), data=dfDM_Temp, REML=FALSE)

dffit <- lmer(A1c ~  AGE + Study + (1 +bs(A1c_MONTHS,df=3) | IP_PATIENT_ID), data=dfDM_Temp, 
REML=FALSE)

KRmodcomp(dffit,dffitR)

large : A1c ~ AGE + Study + (1 + bs(A1c_MONTHS, df = 3) | IP_PATIENT_ID)
small : A1c ~ Study + (1 + bs(A1c_MONTHS, df = 3) | IP_PATIENT_ID)
```

```
          stat     ndf      ddf F.scaling  p.value
Ftest   6.839   1.000 184.104         1 0.009658 **
---
Signif. codes:  0 '***' 0.001 '**' 0.01 '*' 0.05 '.' 0.1 ' ' 1
```

Testing for the statistical significance of study group.

```
dffitR <- lmer(A1c ~ AGE + (1 +bs(A1c_MONTHS,df=3) | IP_PATIENT_ID), data=dfDM_Temp, REML=FALSE)

dffit <- lmer(A1c ~  AGE + Study + (1 +bs(A1c_MONTHS,df=3) | IP_PATIENT_ID), data=dfDM_Temp,
REML=FALSE)

KRmodcomp(dffit,dffitR)

large : A1c ~ AGE + Study + (1 + bs(A1c_MONTHS, df = 3) | IP_PATIENT_ID)
small : A1c ~ AGE + (1 + bs(A1c_MONTHS, df = 3) | IP_PATIENT_ID)
           stat      ndf      ddf F.scaling  p.value
Ftest   9.8966   1.0000 201.9378         1 0.001906 **
---
Signif. codes:  0 '***' 0.001 '**' 0.01 '*' 0.05 '.' 0.1 ' ' 1
```

sFigure 6. Plot fitted A1c data

```
preds <- predict(dffit, newdata=dfDM_Temp)

ggplot(dfDM_Temp, aes(A1c_MONTHS, preds, color = factor(IP_PATIENT_ID))) +     # plot individual
BMIs and spline fits per patient
  geom_line(show.legend=FALSE) +
  geom_point(data = dfDM_Temp, aes(y = A1c)) +
  guides(color = guide_none()) +
  labs(y="Observed (Points) and Predicted (Lines) A1c",x="Months Afer Surgery" )
```

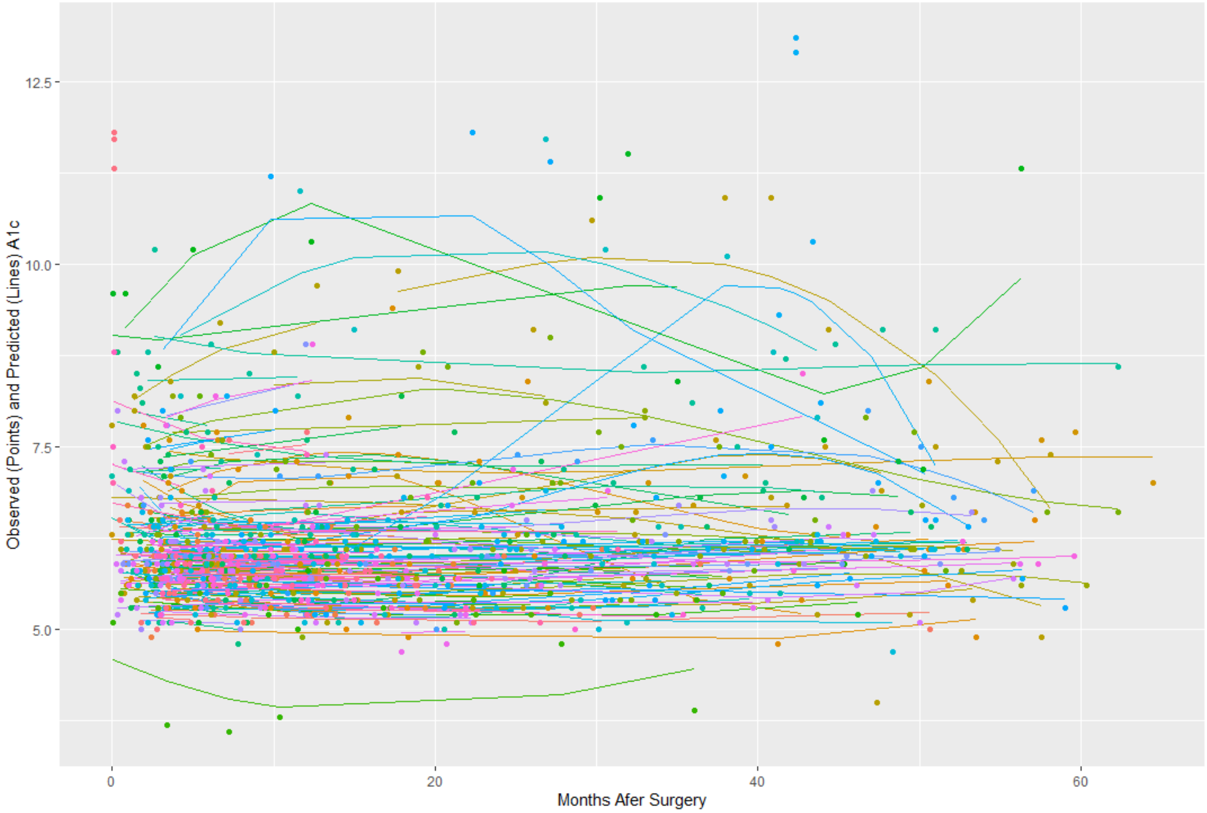

Fitted values for A1c v time after surgery are not as close as were those for BMI v time.

sFigure 7. Modeled A1c After Bariatric Surgery-Stratify by No RCT Criteria v DSS

```
ggplot(dfDM_Temp, aes(A1c_MONTHS, A1c) ) +
  geom_point() +
  stat_smooth(data=subset(dfDM_Temp,Study=='Neither'),color='green',method="lm",formula = y ~
bs(x,3)) +

  stat_smooth(data=subset(dfDM_Temp,Study=='STAMPEDE'),color='red',method="lm",formula = y ~
bs(x,3))
```

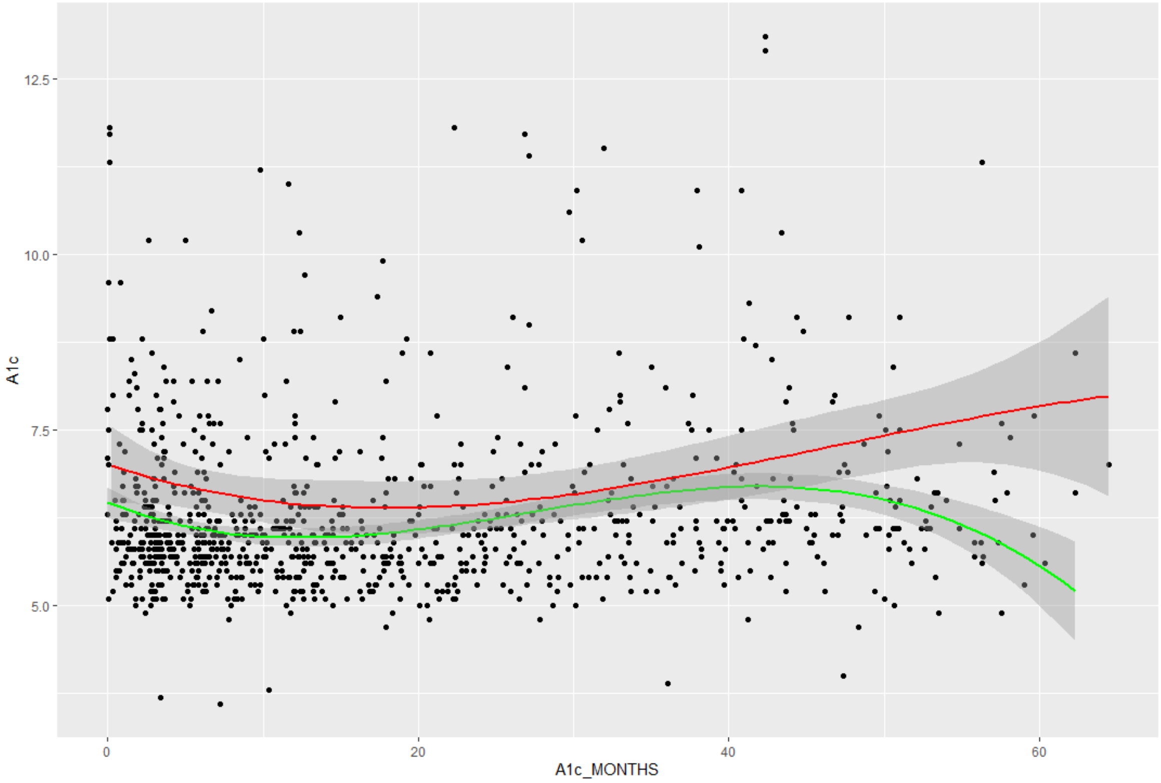

The STAMPEDE group (red line) had higher initial A1c levels and were, on average, higher than the non-RCT eligible patients (green line) throughout the follow up period.
